# Serial case report of high seizure threshold patients that responded to the lengthening of pulse width in ECT

**DOI:** 10.1002/npr2.12224

**Published:** 2021-12-24

**Authors:** Hiroshi Katagai, Norio Yasui‐Furukori, Hirotsugu Kawashima, Taro Suwa, Chieko Tsushima, Yoshiteru Sato, Kazutaka Shimoda, Hiroichi Tasaki

**Affiliations:** ^1^ Department of Neuropsychiatry Hirosaki‐Aiseikai Hospital Hirosaki Japan; ^2^ Department of Psychiatry Dokkyo Medical University School of Medicine Tochigi Japan; ^3^ Department of Psychiatry Graduate School of Medicine Kyoto University Kyoto Japan

**Keywords:** “long” brief pulse waves, electroconvulsive therapy, seizure thresholds, thymatron

## Abstract

Electroconvulsive therapy (ECT) has been used as an effective treatment modality for psychiatric disorders. In patients with high seizure thresholds, augmentation strategies are considered such as changing anesthetic agents, hyperventilation, and premedication with theophylline. We tried to switch to “long (1.5 ms)” brief pulse ECT in all six patients from October 2020. The successful induction of effective seizures with “long” brief pulse stimulation in five of six patients who could not be treated adequately with standard ECT. In the current situation in cases in which brief pulse ECT, with the maximum dose did not lead to effective seizures, “long” brief pulse waves may be a promising option.

## Abstract 2

We tried to switch to “long (1.5ms)” brief pulse ECT in all six patients who had high seizure threshold with standard ECT. The successful induction of effective seizures with “long” brief pulse stimulation in five of six patients.

## Graphical abstract

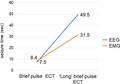

## 1 INTRODUCTION

Electroconvulsive therapy (ECT) has been used as an effective treatment modality for psychiatric disorders, including depression and treatment‐resistant schizophrenia. However, we often experience cases in which brief pulse ECT, with the maximum dose of Thymatron^®^, fails to induce effective seizures. The problem of these “difficult to treat” cases is more serious in some countries, including Japan, where the maximum stimulation dose of Thymatron^®^ is limited to 504 mC. In such patients with high seizure thresholds, augmentation strategies are considered, including changing anesthetic agents, hyperventilation, premedication with theophylline, and so on.^1^

In addition to the abovementioned seizure augmentation methods, modification of stimulus parameters has recently been proposed. Lower frequency, longer stimulation time^2^ and shorter pulse width^3^, ^4^ are thought to be advantageous factors for seizure induction. Ravishankar et al reported a case in which effective seizure induction was achieved by decreasing the pulse frequency and increasing the duration of stimulation while using the same amount of electrical stimulation in a patient with no or inadequate seizures.^5^ On the other hand, Inomata et al reported that seizures could be induced by extending the pulse width to 1 ms or 1.5 ms in patients who could not be induced by 100% dose with low 0.5 and low 0.25 regimens of Thymatron^®^.^6^ Kawashima et al also found improvements in the seizure waveform by lengthening the pulse width.^7^ Here, we report six cases of successful switching to “long” brief pulse (1.5 ms) wave therapy in patients in whom no seizures were induced by standard brief pulse ECT with a 0.5 ms pulse width.

## 2 METHODS

This case‐series study was approved by the Ethics Committee of Hirosaki‐Aiseikai Hospital. Written informed consent was obtained from each patient. The subjects were six individuals with treatment‐resistant schizophrenia or major depressive disorder (Table 1). A Thymatron^®^ System IV (Somatics LLC) and the LOW 0.5 program were used in accordance with the manufacturer's instructions. The minimum duration for an adequate seizure was 15 seconds in the electroencephalogram record of the Thymatron^®^ stimulator. These patients were unresponsive to appropriate and sufficient amounts of medication and had been treated with standard modified ECT, who did not develop adequate seizures with 504 millicoulombs (mC) bilateral electrical stimulation, which gradually failed to produce therapeutic seizures. All patients underwent ECT with small doses of anesthesia and hyperventilation. Some were treated with additional theophylline 250 mg, but all failed to induce effective seizures.

**TABLE 1:** Patient characteristics and ECT parameters in six patients switched from brief pulse ECT to “Long” brief pulse ECT

	Case 1	Case 2	Case 3	Case 4	Case 5	Case 6
Age (y)	66	39	55	68	72	56
Sex (M/F)	M	M	M	M	M	M
Diagnosis	schizophrenia	schizophrenia	schizophrenia	schizophrenia	depression	schizophrenia
Duration of illness (y)	37	23	29	51	10	12
Before “Long” brief pulse ECT						
Additional treatment	Theophylline 250mg			Theophylline 250mg	Theophylline 250mg	
Pulse Width (mSec)	0.5	0.5	0.5	0.5	0.25	0.5
%Energy Set (%)	100	100	100	100	100	100
Stimulus Duration (sec)	8.0	8.0	8.0	8.0	8.0	8.0
Frequency (Hz)	70	70	70	70	140	70
EEG Endpoint (Sec)	19	0	26	0	0	0
EMG Endpoint (Sec)	N/A	0	20	12	10	0
Postictal Suppression Index (%)	89.5	N/A	81.5	N/A	N/A	96.7
Maximum Sustained Coherence (%)	96.4	N/A	92.5	N/A	70.4	N/A
After “Long” brief pulse ECT						
Pulse Width (mSec)	1.5	1.5	1.5	1.5	1.5	1.5
%Energy Set (%)	100	100	100	100	100	100
Stimulus Duration (sec)	2.7	6.2	6.2	2.7	2.7	4.7
Frequency (Hz)	70	30	30	70	70	40
EEG Endpoint (Sec)	61	19	58	79	45	35
EMG Endpoint (Sec)	35	18	35	36	39	26
Postictal Suppression Index (%)	73	97	93	75	89	97
Maximum Sustained Coherence (%)	99	99	99	98	98	100

## 3 RESULTS

**FIGURE 1:**
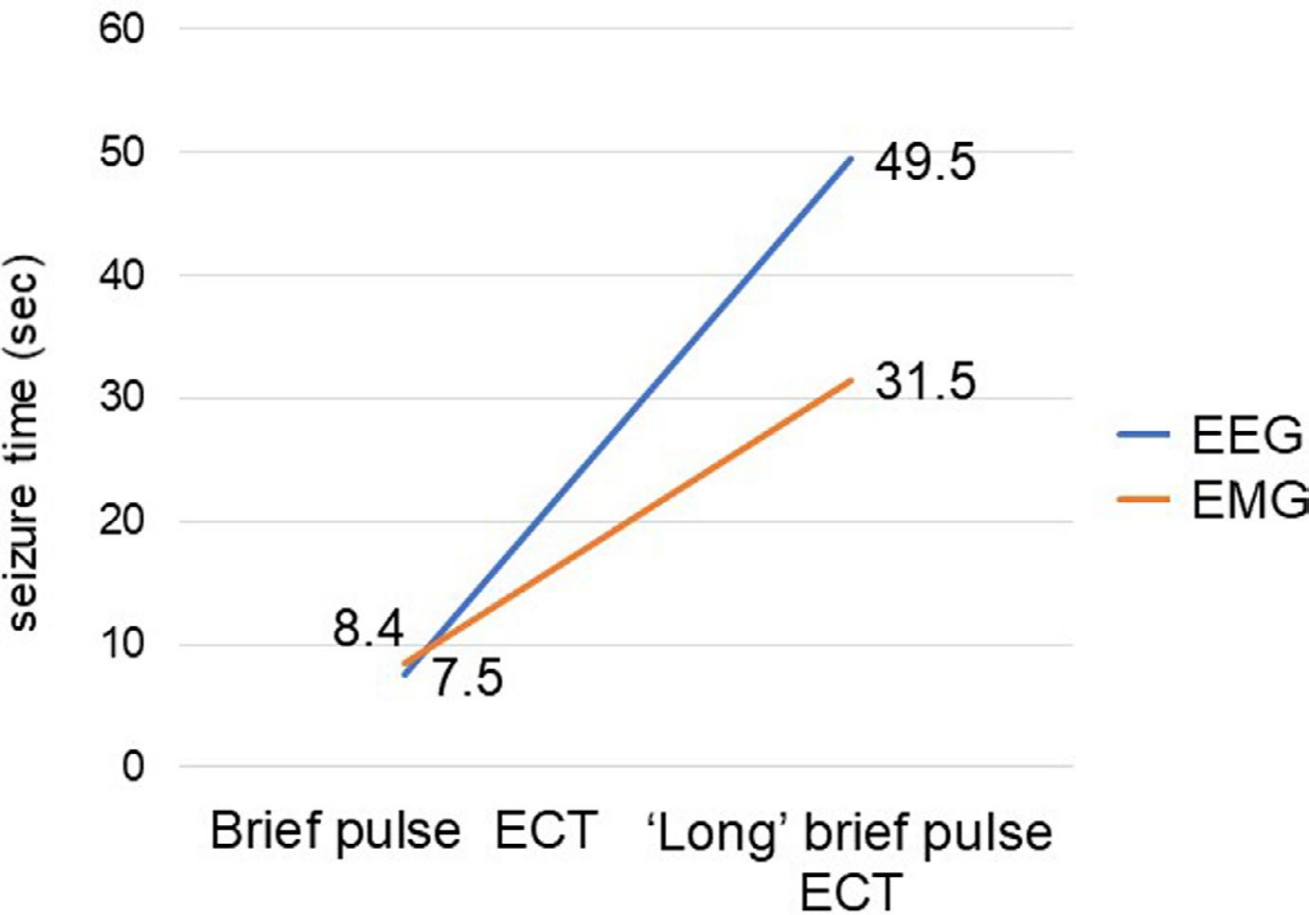
Changes in seizure duration measured by EEG and EMG before and after “long” brief pulse electroconvulsive therapy. Data show mean. Abbreviations: EEG, electroencephalogram; EMG, electromyography. Seizure duration measured by EEG and EMG were both prolonged with “long” brief pulse electroconvulsive therapy

We tried to switch to “long (1.5 ms)” brief pulse ECT in all six patients from October 2020. In the first session, effective seizures were obtained in all patients, without serious adverse effects (Table 1). Seizure duration measured by EEG and EMG were both prolonged with “long” brief pulse electroconvulsive therapy (Figure 1). In the subsequent treatment, five patients continued to undergo successful “long” brief pulse ECT. However, in patient 4, effective seizures were not obtained in the next treatment. No cognitive impairment or other adverse events were observed in any patient after the switch.

## 4 DISCUSSION

The main finding was the successful induction of effective seizures with “long” brief pulse stimulation in five of six patients who could not be treated with adequately standard ECT. In general, shorter pulse widths have lower seizure thresholds. Notably, the effectiveness of long brief pulse stimulation may be attributable to differences in the areas that were depolarized,^8^ and longer pulse widths could stimulate a wider range of brain areas (computer simulation).^9^

Although Swartz and Manly4 concluded that a 0.5 msec pulse width is more efficient than a 1 msec pulse width, there has been some case reports with effective seizures due to lengthening pulse width.^6^, ^7^ Inomata et al used the pulse width fixed at 0.5 ms for a 35‐year‐old schizophrenia patients at first, but this failed to induce seizure at the maximum dose of electrical charge (504 milicoulomb).^6^ They then changed pulse width fixed at 0.25 ms, but this did not induce seizure, either. They then changed pulse width settings to 1.0 ms, which successfully induced seizure with the desirable waveform and of sufficient duration. For the remainder of the treatment, they administered ECT, decreasing the electrical charge to avoid side‐effects. At the 1.0 ms pulse width, they achieved desirable seizures with 60 percent of the maximal charge. At the 1.5 ms pulse width, they succeeded in inducing seizure at 40 percent of the maximal charge. Kawashima et al used the pulse width fixed at 0.5 ms for two elderly depressive patients at first.^7^ Seizure induction failed and they attempted to adjust the pulse width to 1.25 msec or 1.5 msec, which seizure induction was successful.

In the current situation in which brief pulse ECT, with the maximum dose did not lead to effective seizures, “long” brief pulse waves may be a promising option. However, the efficacy and safety of this method has not yet been completely confirmed; therefore, clinical trials with a large number of patients are needed. It is also necessary to examine what kind of frequency and stimulus duration settings with “long” brief pulse waves can produce optimal outcome.

## 5 CONCLUSION

This study suggests the successful induction of effective seizures with “long” brief pulse stimulation in five of six cases in which brief pulse ECT, with the maximum dose did not lead to effective seizures. However, the efficacy and safety of this method has not yet been completely confirmed; therefore, clinical trials with a large number of patients are needed.

## CONFLICT OF INTEREST

The authors declare no conflicts of interest.

## AUTHOR CONTRIBUTIONS

KH and CT were involved in data collection. NYF and KH were involved in data analysis and wrote the first draft of the manuscript. HK, TS, YS, KS, and HT contributed to the interpretation of the data.

## APPROVAL OF THE RESEARCH PROTOCOL BY AN INSTITUTIONAL REVIEW BOARD

This study was approved by the medical Ethics Committee of Hirosaki‐Aiseikai Hospital. The institution was supportive of the publication of case details.

## INFORMED CONSENT

All the patients provided informed consent, and this study was conducted in accordance with the Declaration of Helsinki.

## REGISTRY AND THE REGISTRATION NO. OF THE STUDY/TRIAL

n/a.

## ANIMAL STUDIES

n/a.

## Data Availability

The data that support the findings of this study are openly available in Table 1.
